# Temporal trends in myocardial ischemia risk estimated from 12-lead electrocardiograms using deep learning in individuals with suspected cancer during health checkups

**DOI:** 10.1186/s40959-026-00466-2

**Published:** 2026-03-02

**Authors:** Ken Kurisu, Maiko Fujimori, Kohei Takeshita, Akira Fukui, Kyoko Ito, Keitaro Yokoyama, Tomohiro Kato, Tatsuo Akechi, Kazuhiro Yoshiuchi, Yosuke Uchitomi

**Affiliations:** 1 Department of Cancer Survivorship and Digital Medicine, The Jikei University School of Medicine, Tokyo, Japan; 2 Division of Survivorship Research, National Cancer Center Institute for Cancer Control, Tokyo, Japan; 3 Department of Stress Sciences and Psychosomatic Medicine, Graduate School of Medicine, The University of Tokyo, Tokyo, Japan; 4 Department of Innovation for Medical Information Technology, The Jikei University School of Medicine, Tokyo, Japan; 5 Division of Nephrology and Hypertension, Department of Internal Medicine, The Jikei University School of Medicine, Tokyo, Japan; 6 Center for Preventive Medicine, The Jikei University Hospital, Tokyo, Japan; 7 Harumi Triton Clinic of Jikei University Hospital, The Jikei University School of Medicine, Tokyo, Japan; 8 Department of Endoscopy, The Jikei University School of Medicine, Tokyo, Japan; 9 Department of Psychiatry and Cognitive-Behavioral Medicine, Graduate School of Medical Sciences, Nagoya City University, Nagoya, Japan

**Keywords:** deep learning, cancer, cardiovascular disease, myocardial infarction, psychological distress

## Abstract

**Background:**

Previous studies have suggested the potential effect of psychological stress related to cancer diagnosis on cardiovascular mortality. This study aimed to investigate the temporal trends of cardiovascular risk before and after cancer diagnosis using a deep learning model applied to 12-lead electrocardiograms (ECGs).

**Methods:**

We developed a deep learning model using a publicly available large-scale dataset to quantify myocardial ischemia risk from 12-lead ECGs. We collected ECG records from individuals diagnosed with cancer at a university hospital who also underwent an ECG as part of a health checkup within 90 days prior to cancer diagnosis. The deep learning model was then applied to the ECGs of individuals with cancer, and the temporal trend of cardiovascular risk was examined.

**Results:**

The deep learning model demonstrated high predictive performance, with an area under the receiver operating characteristic curve of 0.930 (95% confidence interval = 0.920–0.941). The model was then applied to 523 ECG records of 89 individuals with cancer. The estimated probability of ECG-indicated myocardial ischemia increased until cancer diagnosis, peaked shortly after diagnosis, and then declined.

**Conclusions:**

These findings support the immediate effect of psychological stress related to cancer diagnosis on increased cardiovascular risks.

**Supplementary Information:**

The online version contains supplementary material available at 10.1186/s40959-026-00466-2.

## Background

Population-based studies have shown that individuals diagnosed with cancer have an elevated risk of cardiovascular mortality [1]. This risk peaks immediately after diagnosis and then declines, following a trajectory similar to that of suicide [2, 3]. This implies that psychological stress related to cancer diagnosis may contribute to the onset of cardiovascular disease. Psychological stress reportedly affects the onset and progression of cardiovascular disease [4, 5]. A bidirectional relationship has been observed between cardiovascular disease and psychiatric conditions such as depression and post-traumatic stress disorder (PTSD) [6, 7]. Nevertheless, other factors, including treatment-related cardiotoxicity, are associated with cardiovascular disease in individuals with cancer [8, 9]. We hypothesized that a sharp increase in cardiovascular risk around the time of diagnosis would support the effect of psychological stress.

In Japan, 12-lead electrocardiograms (ECGs) are commonly performed during routine health checkups and medical practice. Thus, ECGs may provide pre-diagnostic information on the cardiovascular status of individuals who are later diagnosed with cancer. Deep learning has enabled the estimation of various characteristics from 12-lead ECGs, including body weight [10, 11], age, sex [12, 13], and depression [14], and has recently been applied to research in cardio-oncology [15]. Deep learning can predict cardiovascular diseases, such as myocardial infarction and arrhythmias, with performance comparable to that of human experts [16, 17]. We reasoned that this approach could transform binary outcomes, such as the presence or absence of ECG abnormalities, into continuous probabilities of cardiovascular abnormalities. This transformation may enhance the statistical power for risk changes, even when the number of cardiovascular events is limited among datasets.

This study aimed to investigate the temporal trends in cardiovascular risk before and after cancer diagnosis using deep learning applied to 12-lead ECGs. This analysis would support the effect of psychological stress related to cancer diagnosis on the onset of cardiovascular diseases.

## Methods

### Deep learning model for cardiovascular risk

We first developed a deep learning model to quantify cardiovascular risk from 12-lead ECGs using the PTB-XL dataset, which is a publicly available dataset containing 12-lead ECG recordings and corresponding diagnostic labels [18]. The original ECG recordings consisted of 10-second waveforms sampled at 500 Hz. Following the providers’ recommendations, we used folds 1–8 for training (*N* = 17,416), fold 9 for validation (*N* = 2,183), and fold 10 for testing (*N* = 2,198).

The PTB-XL dataset includes labels indicating the following diagnostic superclass: ST-T changes, myocardial infarction, conduction disturbance, hypertrophy, and normal [18]. Each ECG can have multiple labels along with rhythm information. All labels were ultimately confirmed by human cardiologists. In the present study, deep learning models were trained to classify an ECG into two categories: (a) either ST-T changes or myocardial infarction, and (b) any other diagnostic superclass.

Because no model has been widely applied to one-dimensional ECGs in previous studies, we used the same structure as that reported in a previous study [10], comprising three one-dimensional convolutional layers followed by three fully connected layers (Supplementary Fig. 1).

AdamW with a cross-entropy loss was used as the optimizer. We tuned hyperparameters, including ECG waveform length (2–3 s), sampling frequency (100–250 Hz), batch size (128 or 256), learning rate (0.01, 0.005, or 0.001), weight decay (0.005 or 0.001), and number of epochs (25 or 50), using the validation dataset. Based on a prior study using the same architecture that achieved good performance with short waveform segments [10], waveform length and sampling frequency were explored within downsized ranges. Multiple combinations of these hyperparameters were explored, and the combination yielding the highest area under the curve (AUC) of the receiver operating characteristic curve on the validation dataset was selected. The final model performance was then evaluated using the test dataset.

To calibrate the output of the trained model, we applied locally estimated scatterplot smoothing (LOESS) using the model predictions on the training set and their corresponding labels. The span parameter was set at 0.5. This calibration enabled us to interpret the model output as the probability of either an ST-T change or myocardial infarction [16].

These analyses were performed using Python 3.9.18 and PyTorch 2.1.0.

### Evaluation of features contributing to model predictions

To examine generalized differences across superclass labels, we trained an unsupervised autoencoder, with an encoder architecture similar to the main model, to reconstruct the input ECG waves. The latent representation was a 32-dimensional vector (Supplementary Fig. 2), and its distribution was then visualized using the Uniform Manifold Approximation and Projection (UMAP) on the test dataset. Furthermore, the association between each latent vector component and the abnormality (ST-T changes or myocardial infarction) was evaluated using multiple logistic regression.

To further assess whether the deep learning model captures clinically relevant features, we performed the following analyses on the test dataset. First, we examined the distribution of model predictions for ECGs with abnormalities other than ST-T changes or myocardial infarction. In addition, to account for potential confounders, multiple logistic regression was applied to examine the association between model prediction and the abnormalities, adjusting for age, sex, weight, facility, and device. Finally, the final convolutional layer output was visualized using the gradient-weighted class activation map (Grad-CAM).

### Data collection from individuals with suspected cancer during health checkups

Inclusion criteria were as follows: (a) individuals aged ≥ 18 years who were diagnosed with cancer at The Jikei University Hospital between April 1, 2014 and August 31, 2024; (b) those who underwent ECG recording as part of a health checkup at The Jikei University affiliated facilities (Center for Preventive Medicine or Harumi Triton Clinic); and (c) ECGs recorded within 90 days prior to cancer diagnosis.

The timing of cancer diagnosis was defined as the date of disease registration in the electronic medical record following the initial hospital visit. Classification of cancer type was based on the International Classification of Diseases, 10th Revision codes and protocols used in previous studies using the National Cancer Registry of Japan [2, 19].

### Statistical analysis

The deep learning model developed using the PTB-XL dataset was applied to ECGs from eligible individuals with cancer at The Jikei University Hospital. The model outputs were calibrated using the LOESS model, providing a probability of ST-T changes or myocardial infarction for each ECG.

Each individual had multiple ECG records, typically one at the time of the health checkup before diagnosis and another after diagnosis at the hospital. To account for repeated measurements, we used a multilevel model with random intercepts. The model included only an intercept and a categorical variable representing the time period. The time variable was divided into nine categories based on previous literature [2], with the period of > 1 year before the diagnosis set as the reference.

These analyses were conducted using R version 4.4.2 and the “lmerTest” package version 3.1-3.

## Results

### Deep learning model

Hyperparameter tuning of the deep learning model developed using the PTB-XL dataset improved the validation AUC from 0.921 in the initial configuration to 0.930 in the final model. The model achieved the best validation AUC with a 3-second duration, 100 Hz sampling rate, batch size of 256, learning rate of 0.005, weight decay of 0.001, and 25 epochs.

**Fig. 1:**
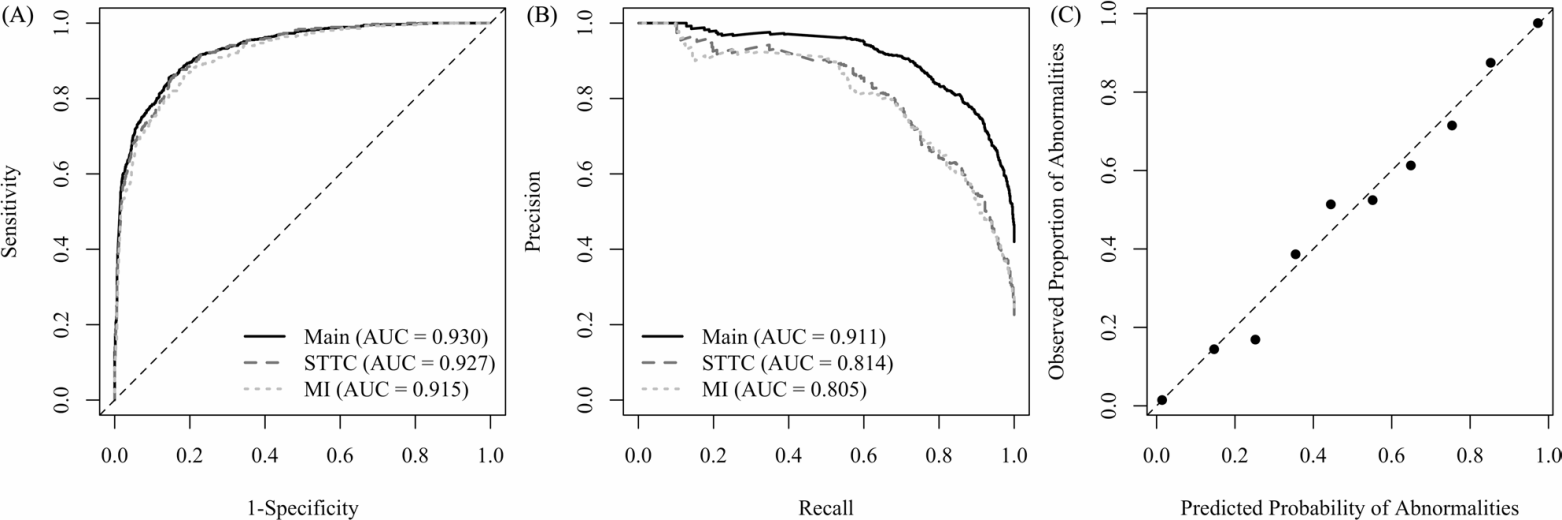
Receiver operating characteristic curves, precision-recall curves, and calibration plot of the deep learning model for predicting ischemic status from 12-lead electrocardiograms. **A **Receiver operating characteristic curves for the combined ischemic outcome (main), ST-T changes (STTC), and myocardial infarction (MI). The curve for STTC was generated by excluding MI cases from the test dataset, and the curve for MI was generated by excluding STTC cases. **B **Precision-recall curves for the same outcomes. **C **Calibration plot of the model output. The dots indicate the observed proportion of abnormalities within each of the 10 predicted probability bins. The dashed line represents the ideal reference line

On the test dataset, the model demonstrated high accuracy in predicting ST-T changes and myocardial infarction, with an AUC of 0.930 (95% confidence interval [CI] = 0.920–0.941), sensitivity of 0.856 (95% CI = 0.833–0.879), and specificity of 0.855 (95% CI = 0.835–0.874) at the optimal cutoff using the Youden index (Fig. 1A).

When ST-T changes alone were used as the outcome (i.e., myocardial infarction cases were excluded from the test set), the AUC was 0.927 (95% CI = 0.912–0.941). Conversely, using myocardial infarction alone as the outcome (i.e., excluding ST-T change cases) yielded an AUC of 0.915 (95% CI = 0.900–0.930) (Fig. 1A). In the precision-recall curves (Fig. 1B), the AUCs were 0.911 for the combined ischemic outcome, 0.814 for ST-T changes, and 0.805 for myocardial infarction.

Figure 1C shows a calibration plot of the model. Calibrated scores were divided into 10 groups based on their values, with each group covering a 0.1 range between 0 and 1. For each group, the mean predicted probability and the mean observed proportion of abnormalities were shown. All 10 points were close to the ideal line, indicating that the model outputs accurately reflected the true probability of an abnormality.

### Features related to deep learning model predictions

Reconstructed ECG waveforms from the autoencoder resembled the originals (Supplementary Fig. 3). UMAP showed no distinct clusters separating normal, ischemic (ST-T changes or myocardial infarction), and other abnormal ECGs (Supplementary Fig. 4). Several latent components were significantly associated with ischemic status. These findings suggest that ischemic ECGs exhibit no clear generalized differences from other ECGs, although latent features may contribute to the model predictions.

Predictions for normal and non-ischemic abnormal ECGs mostly clustered below 0.1 (Supplementary Fig. 5), implying that the model is unlikely to focus on non-ischemic abnormalities. In the logistic regression analyses, the deep learning prediction was strongly associated with the outcome both before (coefficient = 7.92; 95% CI = 7.32–8.55; *P* < 0.001) and after adjustment for potential confounders (coefficient = 9.09; 95% CI = 7.87–10.43; *P* < 0.001), with only small changes in the coefficient. Only age showed a small association (coefficient = 0.05; 95% CI = 0.03–0.07; *P* < 0.001), whereas other covariates (sex, weight, facility, and device) were not significantly related. Furthermore, Grad-CAM heatmaps for ECGs with ST-T changes and myocardial infarction highlighted the QRS complexes and ST segments (Supplementary Fig. 6), which are clinically relevant features.

Overall, these analyses suggest that the deep learning model predicts the outcome based on clinically relevant features rather than generalized or unrelated abnormalities.

### Temporal trends in the relative probability of ECG-indicated myocardial ischemia

A total of 89 individuals underwent an ECG during a health checkup within 90 days prior to their cancer diagnosis, and 523 ECG records were available from these individuals. Their characteristics are summarized in Table 1. Median age of the participants was 52 years, and 43 (48.3%) of them were male.

**Table 1:** Descriptive Statistics

	N = 89
Age, median (range)	52 (22–83)
Male sex, n (%)	43 (48.3)
Body mass index (kg/m²), median (range)	21.9 (16.8–41.2)
Blood pressure (mmHg), median (range)	
Systolic	119 (83–158)
Diastolic	71 (37–103)
Laboratory test results, median (range)	
Blood glucose (mg/dL)	93 (60–324)
Creatinine (mg/dL)	0.71 (0.43–2.90)
LDL cholesterol (mg/dL)	128 (53–203)
Medications, n (%)	
Antihypertensive agents	10 (11.2)
Antidiabetic agents	4 (4.5)
Lipid-lowering agents	7 (7.9)
Medical history, n (%)	
Stroke	0 (0.0)
Heart disease	2 (2.2)
Smoker, n (%)	11 (12.4)
Alcohol, n (%)	
Everyday	6 (6.7)
Sometime	54 (60.7)
None	29 (32.6)
Primary tumor site	
Esophagus	2 (2.2)
Stomach	3 (3.4)
Colon	2 (2.2)
Liver	1 (1.1)
Lung	2 (2.2)
Prostate	1 (1.1)
Bladder	2 (2.2)
Blood	2 (2.2)
Other	74 (83.1)

**Fig. 2:**
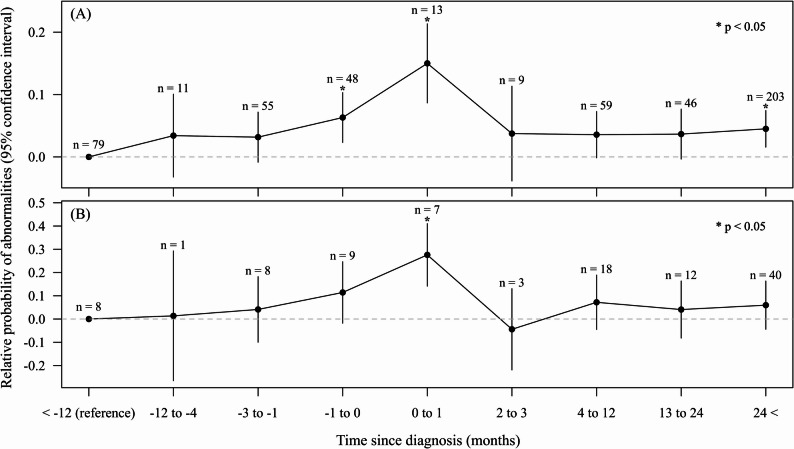
Temporal changes in the relative probability of ECG-indicated myocardial ischemia based on a multilevel model. Panel (**A**) shows results for all participants, and panel (**B**) shows results from a model excluding individuals whose cancer types were classified as others. The dots indicate fixed effect estimates for each time category, shown with 95% confidence intervals. Numbers above the intervals represent the number of ECG recordings. Asterisks denote statistical significance relative to the reference category, defined as recordings from > 12 months before diagnosis

We developed a multilevel model using these data and plotted the fixed-effect coefficients for each time-period category, with the period of > 1 year before the diagnosis set as the reference (Fig. 2A). The estimated probability of ECG-indicated abnormalities increased until diagnosis, peaked immediately after diagnosis, and then decreased. Nevertheless, the estimates remained elevated compared with those during the reference period.

In the primary tumor site classification, the other category accounted for > 80% of the participants (Table 1), and this category included benign tumors [2, 19]. Therefore, we conducted a subgroup analysis excluding patients classified as others, resulting in 106 ECGs from 15 individuals. The time trend observed in this subgroup, shown in Fig. 2B, resembles that shown in Fig. 2A. Additionally, a binary variable representing other tumor types was included in the multilevel model using the entire dataset. This variable was not significantly associated with ECG abnormality scores (fixed effect = 0.043; 95% CI = − 0.031 to 0.116; *P* = 0.24).

## Discussion

In this study, we trained a deep learning model to predict the probability of ST-T changes or myocardial infarction from 12-lead ECG data using a publicly available dataset. We then applied this model to ECGs obtained from individuals with cancer for whom pre-diagnosis ECG data were available to examine temporal changes in the risk of myocardial ischemia. The analysis revealed that, compared with the period of > 1 year before diagnosis, the estimated ischemic risk gradually increased before the diagnosis and peaked immediately after the diagnosis. The risk then declined, although it remained elevated when compared with the reference value.

The peak risk immediately after diagnosis is consistent with previous studies using data from the National Cancer Registry and other sources, which have shown an increased risk of cardiovascular mortality shortly after diagnosis [2, 3]. Moreover, the risk scores were already elevated one month prior to the diagnosis, which also aligns with previous studies that suggest the risks of psychiatric disorders and cardiovascular disease increase even during diagnostic workup [20–22]. Furthermore, ischemic risk increased from just before the diagnosis to immediately after, implying the involvement of psychological distress triggered by the cancer diagnosis. This trend around the time of diagnosis contributes to the literature on the role of psychological stress in cardiovascular disease.

These findings underscore the potential importance of addressing psychological stress to prevent cardiovascular mortality, particularly during the period immediately following cancer diagnosis. Potential interventions include physician communication training aimed at improving psychological support for cancer diagnosis [23]. Additionally, interventions to prevent PTSD after a life-threatening medical event, such as cognitive behavioral therapy, have been explored [24], and such approaches may be applicable in this context. As psychological stress appears to increase before diagnosis, systems should be established to identify high-risk individuals and provide psychological support during the diagnostic process. Efforts have also been made to predict cardiovascular mortality risks during cancer survivorship [25, 26], and similar approaches could be extended to the peri-diagnostic period. Given the common use of 12-lead ECGs in health checkups in Japan, they may be useful in identifying high-risk individuals in need of psychological support during diagnostic evaluation. Further prospective pilot studies are warranted to explore the effectiveness of interventions and preventive strategies.

Nevertheless, the underlying mechanisms linking stress and cardiovascular disease remain largely unclear, although involvement of the hypothalamic–pituitary–adrenal axis and immune responses has been suggested [4, 7]. Recent studies have identified molecular signaling pathways involved in the effect of stress on the cardiovascular system [27] and myocardial dysfunction in PTSD-like mice [28]. Further investigation is needed to clarify the biological mechanisms underlying the association between stress related to cancer diagnosis and cardiovascular disease.

This study has several limitations. First, the PTB-XL dataset does not provide sufficient information on the timing of myocardial infarction relative to the ECG acquisition [18], and the labels for ST-T changes and myocardial infarction may not necessarily reflect clinically diagnosed ischemic conditions. Second, a within-subject comparison of the ECGs before and after diagnosis was not feasible because of the limited sample size. Third, we were unable to evaluate the potential effect of cancer stage or treatment course. Fourth, the date of cancer diagnosis was based on disease registration in the electronic medical records, which may not precisely align with the actual date of diagnosis disclosure. Fifth, psychosocial factors and history of psychiatric disorders were not included in this analysis. Finally, the reliability of risk scores based on the deep learning model was not evaluated for actual cardiovascular events or mortality among individuals diagnosed with cancer. Nevertheless, false-positive rates for myocardial infarction based on ECG ST-elevation are relatively low [29], and ECG abnormalities are associated with future cardiovascular events [30]. Given these limitations, the risk score derived from the model developed should be validated against both cross-sectional and longitudinal ischemic events among individuals with cancer in future studies.

## Conclusions

This study showed that the likelihood of myocardial ischemia gradually increases before cancer diagnosis and peaks immediately after diagnosis. These findings may support the effect of psychological stress related to cancer diagnosis on elevated cardiovascular risk. Further studies are warranted to validate these findings and develop potential interventions.

## Supplementary Information

Supplementary Material 1.

Supplementary Material 2.

## Data Availability

The datasets analyzed in the current study cannot be shared because approval was not obtained from the institutional review board.

## Abbreviations

PTSD: post-traumatic stress disorder
ECG: electrocardiogram
LOESS: locally estimated scatterplot smoothing
AUC: area under the curve
UMAP: Uniform Manifold Approximation and Projection
Grad-CAM: gradient-weighted class activation map
CI: confidence interval
