# Supplementary material for: Temporal trends in myocardial ischemia risk estimated from 12-lead electrocardiograms using deep learning in individuals with suspected cancer during health checkups
Source: Cardiooncology. 2026 Mar 2;12:47. doi: 10.1186/s40959-026-00466-2 (PMC13059618; doi:10.1186/s40959-026-00466-2)
Supplement: Supplementary file 1 — Supplementary Material 1. [file 40959_2026_466_MOESM1_ESM.docx]

**Supplementary Figure 1. Structure of deep learning model**

**
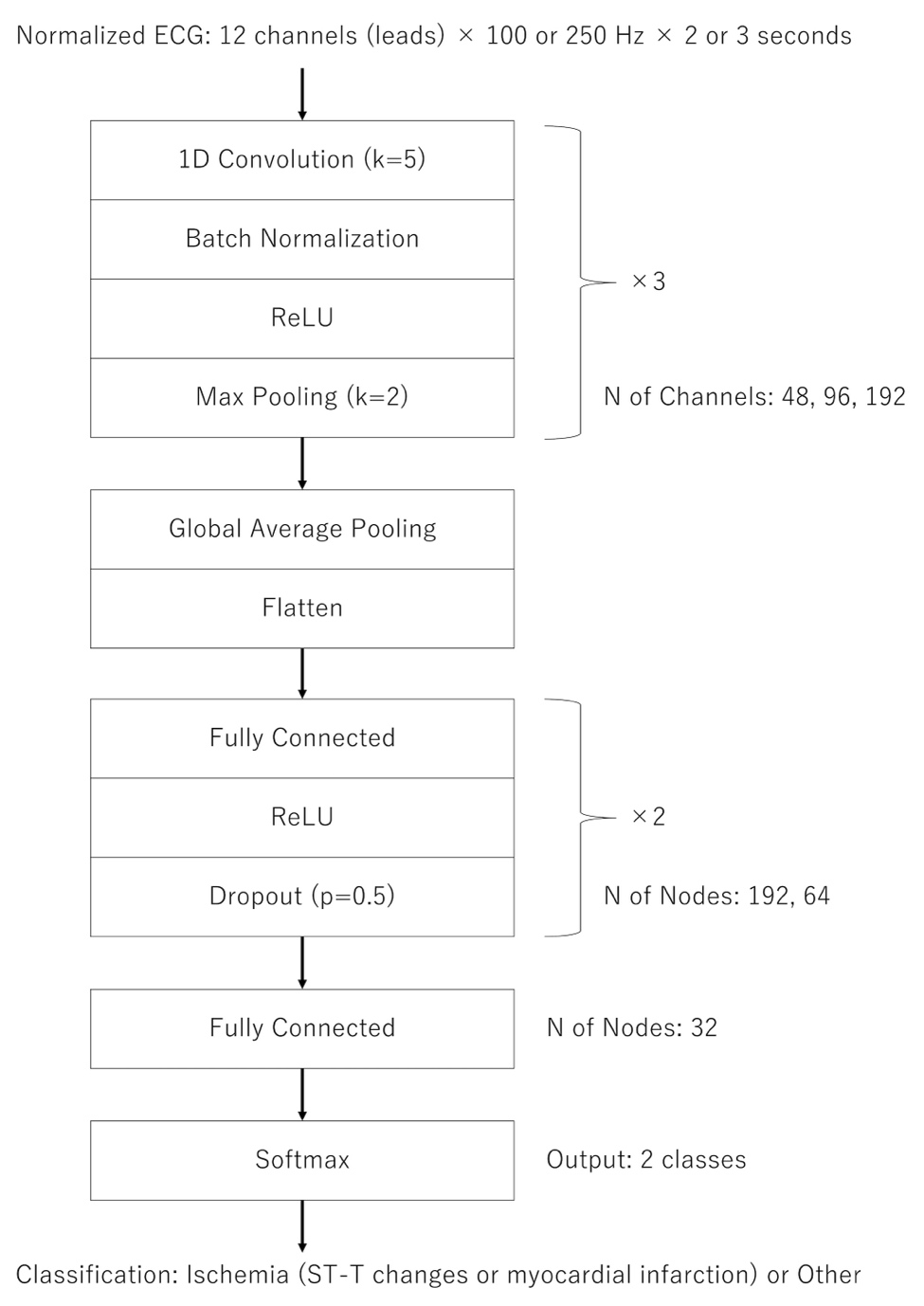
**

The network consists of three blocks of one-dimensional convolutional layers and three blocks of fully connected layers. Kernel size, pooling size, number of channels in the convolution layers, and number of nodes in the fully connected layers are indicated. This architecture is identical to that used in a previous study (Kurisu et al., Sci Rep. 2024;14(1):4696), and the present figure was newly created based on that architecture.

**Supplementary Figure 2. Structure of autoencoder model**


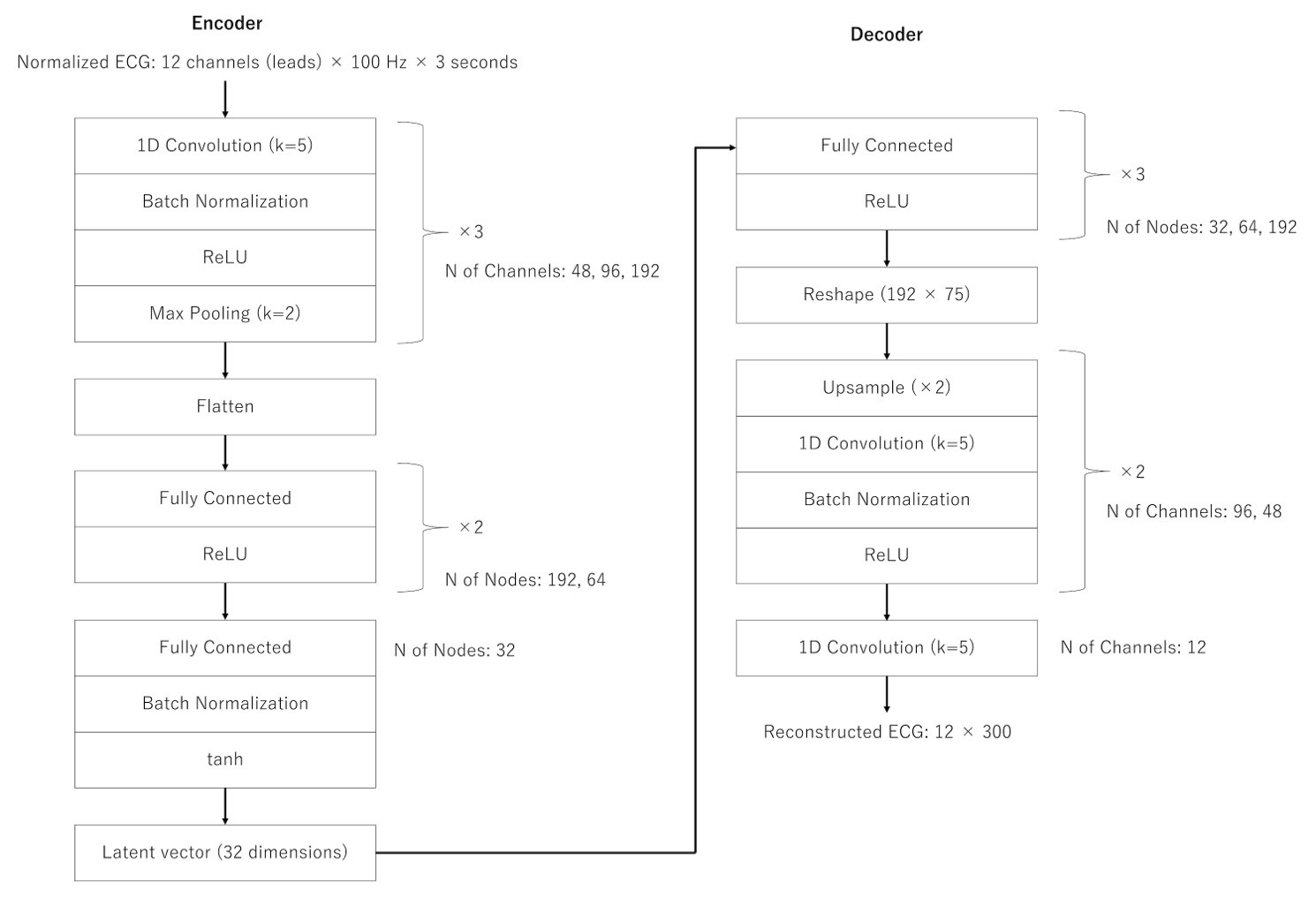


The encoder has a structure similar to the main CNN model and outputs a 32-dimensional latent vector. The decoder takes this latent vector as input, processes it through three blocks of fully connected layers and three blocks of one-dimensional convolutional layers, and reconstructs the ECG. The model was trained to minimize the mean squared error between input and reconstructed ECGs, using the same hyperparameters as the main CNN model.

**Supplementary Figure 3. Example of input and output waveforms of autoencoder**


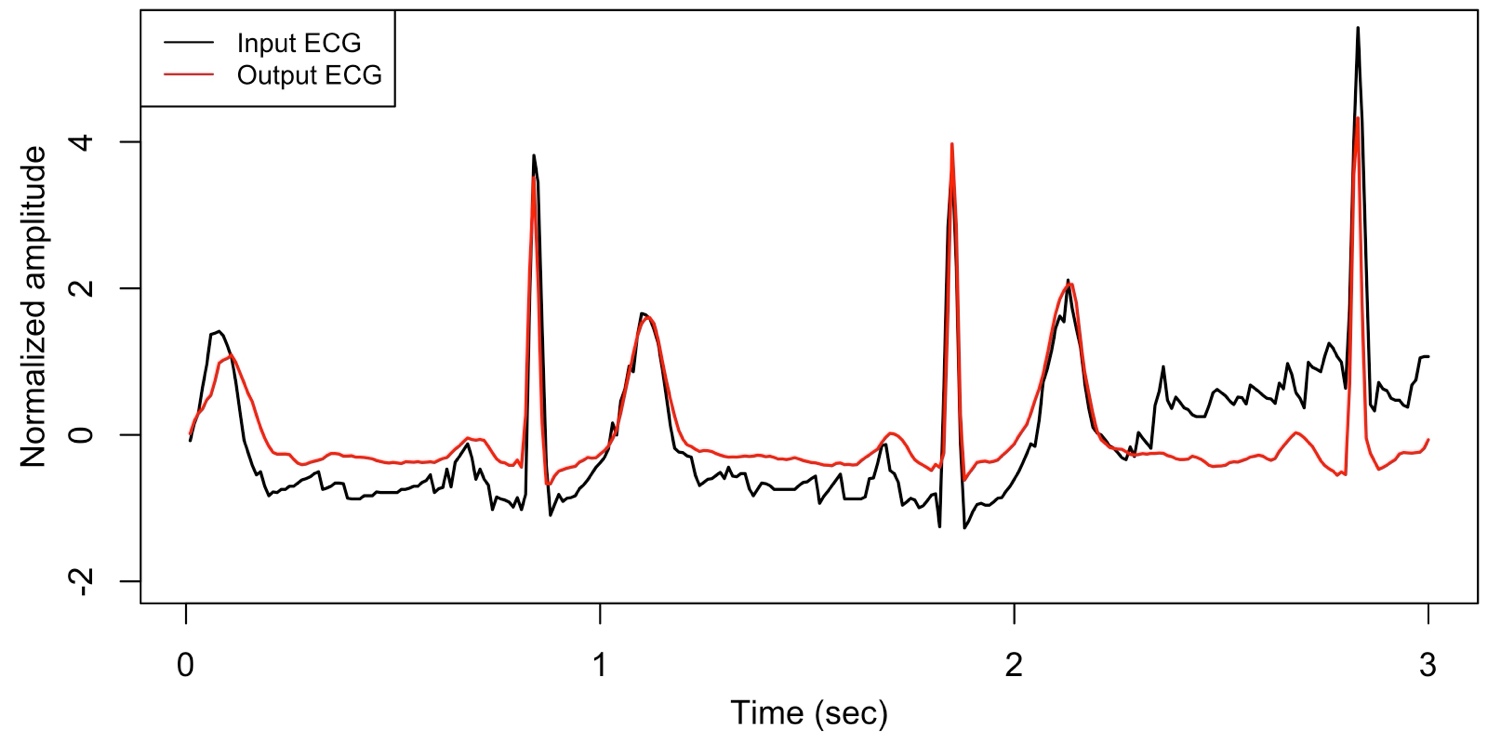


The black line shows an example of input ECG waveform, while the red line shows the waveform reconstructed by the autoencoder model.

**Supplementary Figure 4. UMAP visualization of the latent representation generated by the autoencoder**


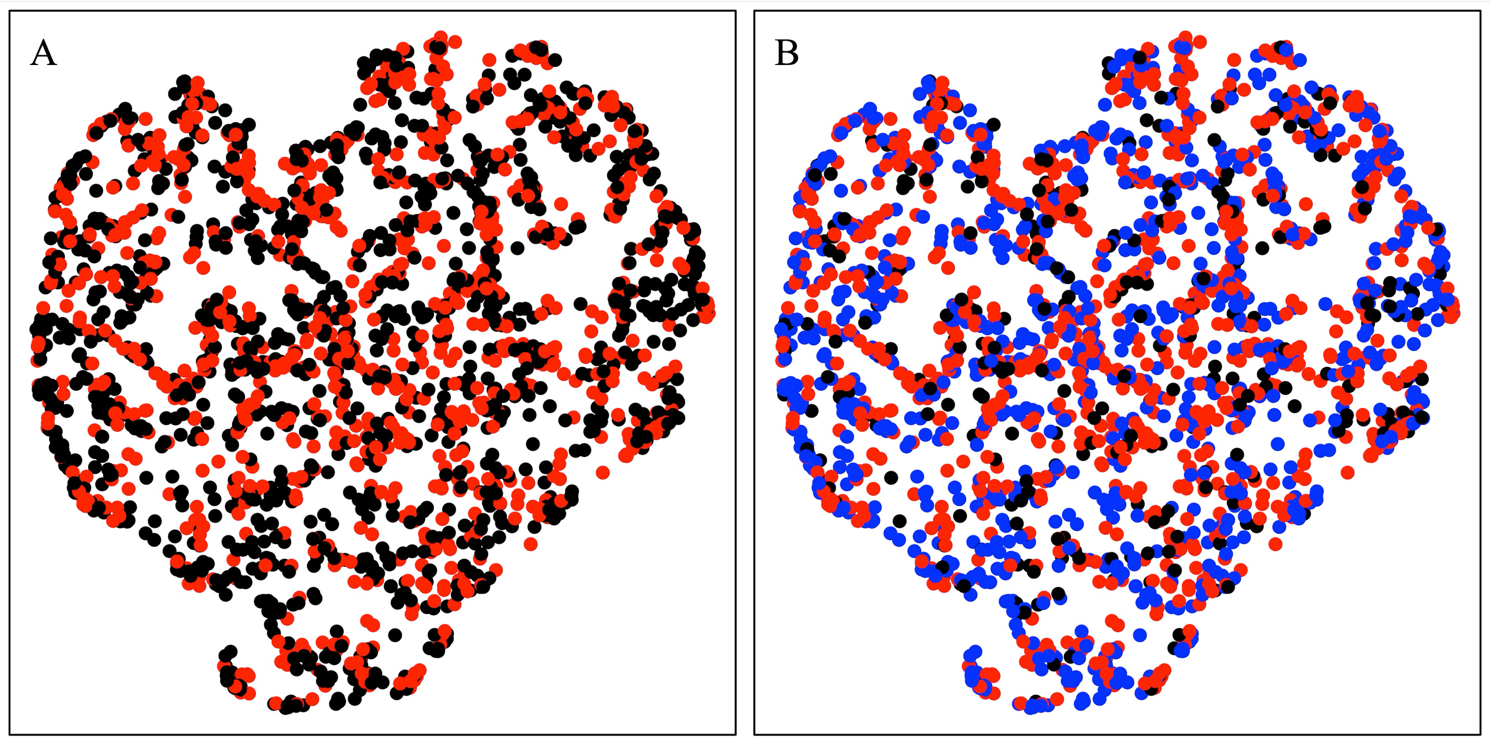


A: Red dots represent ECGs with ischemic findings (ST–T changes or myocardial infarction), and black dots represent all other ECGs. One of the two components was significantly associated with ischemic findings (P = 0.031).

B: Red dots represent ECGs with ischemic findings, blue dots represent ECGs with non-ischemic abnormalities, and black dots represent normal ECGs.

In both panels, the different classes do not exhibit clearly distinct clusters or separations in the latent space.

**Supplementary Figure 5. Distributions of model predictions**


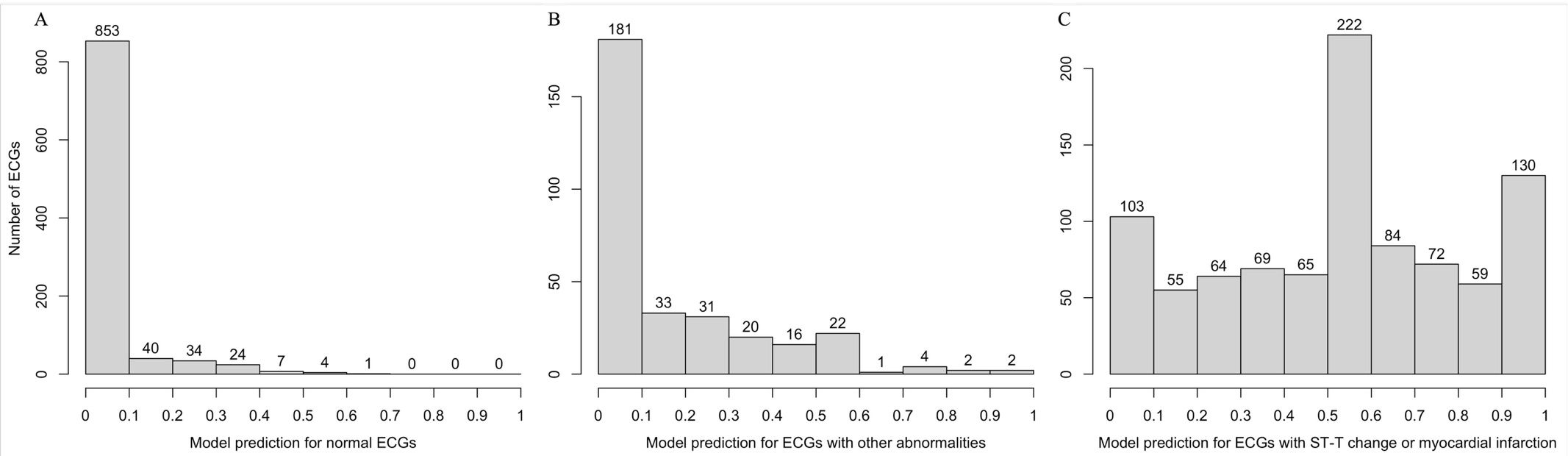


A: Distribution for normal ECGs. Most predictions were concentrated below 0.1.

B: Distribution for ECGs with abnormalities other than ST–T changes or myocardial infarction. Predictions also clustered below 0.1, although a longer tail with higher values was observed compared with normal ECGs.

C: Distribution for ECGs with ST–T changes or myocardial infarction. Predictions were widely distributed across the full range (0.0–1.0).

**Supplementary Figure 6. Examples of Grad-CAM heatmaps**


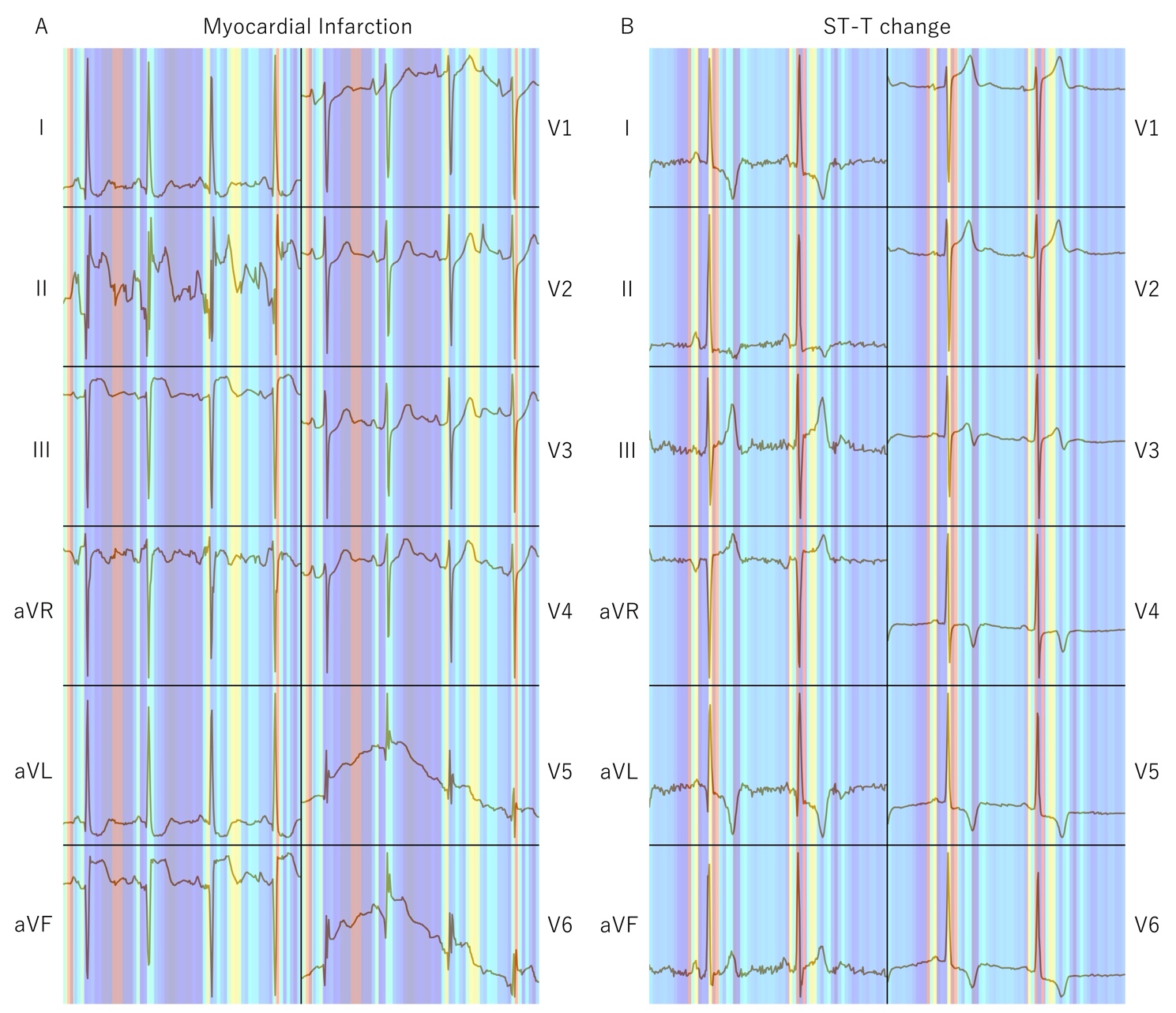


A: Myocardial infarction. B: ST–T changes. Areas in red-to-yellow color greatly influence the prediction, whereas areas closer to blue have minimal impact. In both examples, the model primarily focuses on the QRS complexes and ST segments, which are clinically relevant features for these abnormalities.
